# Extending Specimens to Save Plant DNA: Structuring Department DNA Collections in Times of Biodiversity Loss

**DOI:** 10.1002/ece3.73211

**Published:** 2026-04-08

**Authors:** Claudia González‐Toral, Eduardo Cires

**Affiliations:** ^1^ Royal Botanic Gardens, Kew Richmond UK; ^2^ Department of Organisms and Systems Biology University of Oviedo Oviedo Spain; ^3^ Institute of Natural Resources and Territorial Planning (INDUROT), campus de Mieres Mieres Spain

**Keywords:** biodiversity, conservation, DNA banking, DNA collection, dynamo scheme, extended specimen network

## Abstract

Plant biodiversity DNA banks are scarce despite the current plant biodiversity loss, their value for *ex situ* conservation, DNA preservation progress, and the rapid growth of DNA‐dependent research fields. We explore the principles and basic organization of plant biodiversity DNA banking and propose new ways of addressing the biodiversity loss through their implementation. Small departmental collections can be created through a six‐step holistic process that integrates three types of collections (DNA extracts, DNA‐rich tissues, and herbarium vouchers) to generate extended specimens, thereby contributing to local and global plant biodiversity knowledge and conservation efforts. We propose a revision to the international biobanking strategy (the Dynamo scheme) that leverages the strengths of interconnected small departmental collections. By doing so, we can mitigate the risk of sample loss in the event of a catastrophe, optimize sampling strategies, and provide more comprehensive coverage of taxa and distribution ranges while fostering national and international collaborations.

## Graphical abstract

Small department DNA collections can be created through a six‐step holistic process that integrates DNA extracts, DNA‐rich tissues, and herbarium vouchers to generate extended specimens. We propose a revision to the international biobanking strategy (the Dynamo scheme) that leverages the strengths of interconnected small departmental collections. This mitigates the risk of sample loss, optimizes sampling strategies, and provides more comprehensive coverage of taxa and distribution ranges.

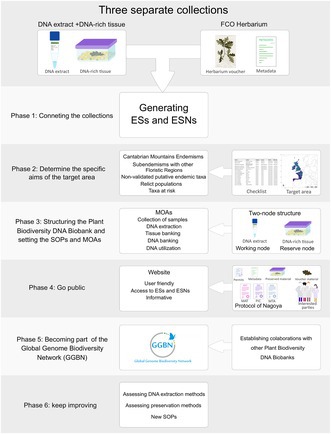

## 1 Introduction

Since the emerge of biological collections three centuries ago, these have become indispensable tools for scientific advances in fields like medicine, taxonomy, ecology, evolutionary biology or genetics (Pyke and Ehrlich 2010; Holetschek et al. 2012; Kang et al. 2013; Martin and Kaye 2000). The first biobank appeared in 1948 in the United States of America (USA) with the establishment of the Framingham Heart Study (FHS) blood bank, a research‐oriented facility designed for the long‐term storage of well‐preserved human specimens and their associated data (Thormann et al. 2006; Kang et al. 2013; Parodi 2015). The advances in preservation technologies resulted in the establishment of many types of biobanks during the following decades (e.g., tissue, disease‐specific or population specific biobanks) (Kang et al. 2013; De Souza and Greenspan 2013; Parodi 2015).

During the late 1960s, international organizations like the Food and Agriculture Organization of the United Nations (FAO) recognized the loss of animal and plant genetic diversity as a pressing conservational issue (Wildt et al. 1993; Heywood 1995). This drew the attention of scientific institutions towards genetic biorepositories over the next two decades, given their potential as *ex situ* conservation tools for genetic diversity preservation (Thormann et al. 2006; León‐Lobos et al. 2012; Seberg et al. 2016; Singh and Singh 2017). Consequently, animal and plant germplasm collections associated with institutions focused on biodiversity conservation, such as museums, zoos, botanic gardens, and university departments, were established (Wilson 1985; Wildt et al. 1993; Krauss et al. 2002). These collections aimed to mitigate the biodiversity loss due to anthropic activities, also known as the Biological Diversity Crisis (BDC), and to ensure the long‐term viability of populations and species (Palacio‐Mejía 2006; De Souza and Greenspan 2013; Leigh et al. 2019). Gradually, these collections evolved into systematized biobanks preserving a range of high‐quality organic material and their associated information. These included germplasm banks (explants storage), seed banks, gene banks (vegetative propagules), tissue banks, pollen banks, or DNA banks (Wildt et al. 1993; Adams 1997; Palacio‐Mejía 2006; Rice, Henry, and Rossetto 2006; Thormann et al. 2006; Astrin et al. 2013; Rajasekharan et al. 2013; De Souza and Greenspan 2013; Dröge et al. 2016; Comizzoli and Wildt 2017; Ruta et al. 2020). Notably, biodiversity DNA biobanks, which focus on wild and/or commercial species, often complement other collections, such as university collections or botanic gardens (Rice, Shepherd, et al. 2006; Jain et al. 2012). Their preserved samples can include DNA libraries, cloned fragments, or complete plant genomes (Organisation for Economic Co‐operation and Development (OECD) 2001; Rice, Shepherd, et al. 2006; Jain et al. 2012; Dröge et al. 2014; Watson 2014; Campbell, Astrin, Brody, et al. 2018).

The DNA technology advances and subsequent cost reductions in the 1990s and 2000s contributed to the widespread use of DNA sequences in phylogenetic and genetic diversity studies, leading to the emergence of department private DNA and/or DNA‐rich plant tissue collections associated to ecological data (Adams 1997; Karp et al. 1997; Jenkins 2003; Rice, Shepherd, et al. 2006; Palacio‐Mejía 2006; Gaudeul and Rouhan 2013; Shaw et al. 2014; Watson 2014; Rabeler 2017). These department private DNA collections paved the way for the development of modern DNA Plant Biodiversity Banks or Biobanks (PBBs), institutions specialized in the long‐term storage and preservation of high‐quality DNA samples, including DNA extracts and DNA‐rich tissue material along with their associated data (Hodkinson et al. 2007; Astrin et al. 2013; Gaudeul and Rouhan 2013). Thus, PBBs have become fundamental tools for addressing the botanical problems arising from the BDC (Blackmore 2002; Thormann et al. 2006; Hodkinson et al. 2007; Astrin et al. 2013; Gaudeul and Rouhan 2013; Dröge et al. 2016). Despite their role in prompting accessibility to high‐quality genomic samples and complementing conservation strategies, as well as the 2000s claims about the proportionally low costs of establishing DNA biobanks on a global scale, PBBs have not been fully integrated into existing biodiversity preservation facilities (Savolainen 2004; Hodkinson et al. 2007; Brown 2011; Astrin et al. 2013).

The BDC has aggravated during the last four decades due to the intensification of processes directly associated to high extinction rates of animals, plants and fungi (De Vos et al. 2015; Eichenberg et al. 2021; International Union for Conservation of Nature (IUCN) 2024) such as habitat loss and fragmentation (e.g., Peres 2001; Mortelliti et al. 2011), the introduction of alien invasive species (e.g., Manenti et al. 2018; Muthukrishnan and Larkin 2020), species overexploitation (e.g., Bodeker et al. 2014; Chuanwu et al. 2019) or changes in land use and Climate Change (e.g., Sekercioglu et al. 2008; Feeley and Silman 2010; De Baan et al. 2013). These synergetic processes are accelerating the extinction rates to 1000 times the background rates, with projections of reaching 10,000 times in the future and no signs of deceleration despite financial crises (De Vos et al. 2015; Steffen et al. 2015). The Intergovernmental Panel on Climate Change (IPCC) projections for terrestrial and freshwater ecosystems are daunting, as estimations suggest a 2°C–4°C rise of global mean temperature, a 35%–40% increase in wildfires and 15%–35% increase in biome shifts, in a context where a 5°C increase would trigger the extinction of 60% of the species inhabiting the affected ecosystems (Parmesan et al. 2022). Currently, 129 plant species have gone completely extinct, 45 are extinct in the wild and over 320,000 flowering plant species are comprised in IUCN Red List of Threatened Species (Antonelli et al. 2023; IUCN 2024). Furthermore, 77% of the estimated 100,000 plant species unknown to science may under extinction threat, yet global risk assessments have only covered about 55% of the threatened red‐listed vascular plants (Nic Lughadha et al. 2020; Antonelli et al. 2023). Additionally, other factors like pollinators abundance decrease could accelerate plants decline, something with potentially devastating and with unforeseeable consequences for ecosystems given plants' fundamental ecosystemic role (Grime 2002; Schleuning et al. 2016; Traveset et al. 2017). Consequently, the synergistic interactions of BDC and Climate Change have economic, social and ecological consequences at global scale making them one main concerns for biodiversity‐focused research (Mace et al. 2014; Urban 2015; Román‐Palacios and Wiens 2020; Kedward et al. 2023; Schlaepfer and Lawler 2023).

The biodiversity loss statistics and risk assessments do not usually take into account the genetic dimension, a relevant aspect for species and population survival as genetic diversity loss is intimately related to extinction debt, which is one of the main issues for plant extinctions estimations (Helm et al. 2009; Garner et al. 2020; Nic Lughadha et al. 2020). Unfortunately, the shortfall in plant biodiversity biobanking (PB biobanking) reported by Hodkinson et al. (2007) persists in a context where biodiversity loss poses an ecological and financial threat to human society due to unpredictable ecosystem effects, insufficient global risk assessments that tend to exclude genetic data and chronic insufficient funding for non‐commercial plants research and conservation (Suárez and Tsutsui 2004; Bradley et al. 2014; Urban 2015; Schleuning et al. 2016; Roberson and Meyer 2020; Kedward et al. 2023). Moreover, despite its potential for boosting basic science and plant conservation, PB biobanking principles have not been fully implemented within the existing biological collections (Hodkinson et al. 2007; Astrin et al. 2013; Gaudeul and Rouhan 2013; Dröge et al. 2014; González‐Toral and Cires 2022). In this context, we aim to explore (1) how PB biobanking principles and activities meet the current scientific and social goals for biodiversity preservation and information sharing and (2) new ways in which the scientific community can address the biodiversity loss through the implementation of PB biobanking principles.

## 2 Material and Methods

We conducted a search of English and Spanish language scientific papers and books in “Google Scholar” (https://scholar.google.com/) and “Web of Science” (https://webofscience.com/wos/) between March 2020 and May 2024. We used the key words: “Biobank,” “DNA Bank,” “Plant Biodiversity,” “DNA preservation,” “biobanking,” “Protocol of Nagoya,” “Access and Benefit‐Sharing,” “Biological Diversity Crisis,” and “Plant Conservation.” We reviewed all relevant articles, reviews, documentation and websites found, regardless of publication date.

### 2.1 PBBs Structure; Functioning and Classic Global Scheme

**FIGURE 1:**
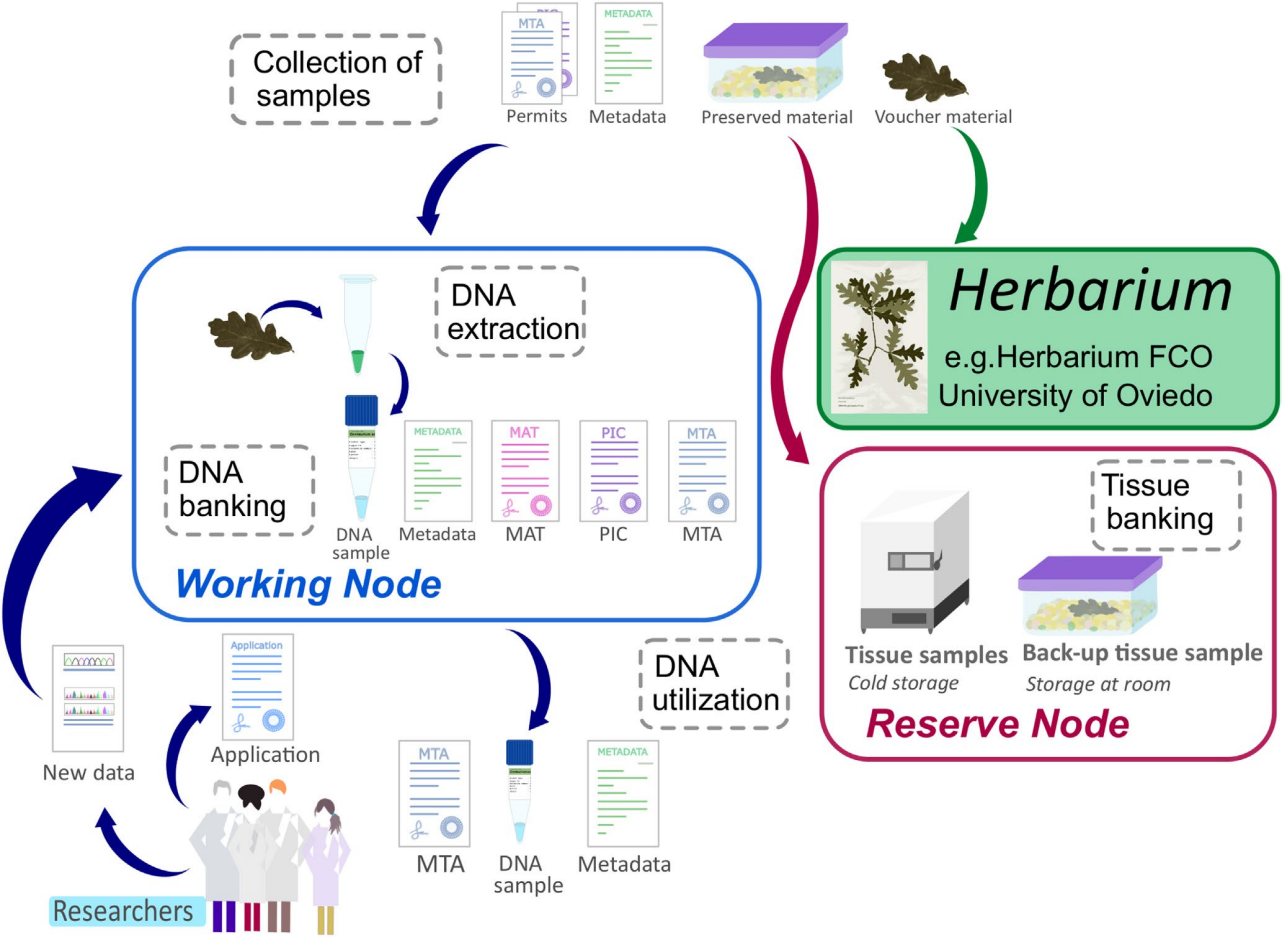
Scheme depicting the main Major Operational Activities (MOAs) of a plant DNA biobanks described as described by Hodkinson et al. (2007) and the main functions of the working and reserved nodes as proposed by Adams (1997) for the DNA bank‐Net. The main MOAs correspond to the name in the dark gray dashed‐line cages, while the working node (WN) and the reserve node (RN) correspond to the blue and red cages respectably.

PBBs are instrumental for the implementation of the Protocol of Nagoya on Access to Genetic Resources and the Fair and Equitable Sharing of Benefits Arising from their Utilization (= Protocol of Nagoya) as well as for supporting conservation strategies and advancing phylogenetic as they provide the framework for access to properly‐managed genomic samples (Blackmore 2002; Hodkinson et al. 2007; Secretariat of the Convention on Biological Diversity (CBD) 2011; Astrin et al. 2013; Dröge et al. 2014; Consortium of Europena Taxonomic Facilities (CETAF) 2020). This is accomplished thanks to their structure and functioning, which are based on a series of “Major Operational Activities” (MOAs) implemented through “standardized operating procedures” (SOPs) (Adams and Adams 1992; Hodkinson et al. 2007; Astrin et al. 2013; Watson 2014). PB biobanking usually involve 5 MOAs: (1) material collection, (2) storage of the plant tissue, (3) DNA extraction, (4) DNA banking, and (5) DNA utilization, integrated in a two‐node structure consisting in a Working Node (WN) and a Reserve Node (RN) (Adams and Adams 1992; Adams 1997; Rice, Shepherd, et al. 2006; Hodkinson et al. 2007; Astrin et al. 2013; Watson 2014; Zimkus and Ford 2014a) (Figure 1). The WN is responsible for sample collection and acquisition, DNA extraction, banking and utilization under controlled conditions (i.e., loans, shipments and interactions with researchers and institutions); meaning that its SOPs are directly related to the Protocol of Nagoya implementation (Adams and Adams 1992; Mattick et al. 1992; Adams 1997; Rice, Henry, and Rossetto 2006; Hodkinson et al. 2007). The RN focuses mainly on tissue banking, usually acts as a WN backup for the WN and/or for other biobanks with different scopes (Adams and Adams 1992; Adams 1997; Caujapé‐Castells et al. 2010; Hodkinson et al. 2007; Zimkus and Ford 2014a; South African National Biodiversity Institute (SANBI) 2020). Samples and metadata are linked through an effective labelling system to ensure specimen traceability (Global Genome Biodiversity Network (GGBN) 2015; Funk et al. 2017; Campbell, Astrin, Brody, et al. 2018; CETAF 2020). PBBs activities through MOAs result in complex systems of interactions, requiring a governance model and structure that ensures transparency to the interested parties and accountability to sample providers, while monitoring the PBB's own functioning (Gottweis and Lauss 2012; Gille et al. 2020).

The advancements in preservation techniques resulted in the establishment of various types of plant biobanks storing different tissues, including: explants under controlled sterile conditions for slow or suspended growth (germplasm biobanks), viable seeds of crops and their wild relatives (e.g., Millennium seed bank), vegetative propagules (gene banks), different types of tissue (e.g., Alexander von Humboldt Institute (AvHI) tissue bank), pollen (e.g., the pollen collection of the ICAR‐National Bureau of Plant Genetic Resources (ICAR‐NBPGR) of India) or DNA extracts (e.g., Kew Royal Botanic Garden) (Palacio‐Mejía 2006; Thormann et al. 2006; Rajasekharan et al. 2013; Ruta et al. 2020). PBBs may focus on certain type of organisms, on a specific scientific sampling strategy or even have specific geographic scope. For instance, the Japanese National Institute of Agrobiological Sciences (NIAS) Genebank (https://www.gene.affrc.go.jp/about_en.php) focuses on agricultural taxa and their wild relatives, while Kew DNA Bank (https://dnabank.science.kew.org/homepage.html) gather samples from wild species (NIAS 2024). The Kostrzyca Forest Gene Bank (https://www.lasy.gov.pl/en/information/news/kostrzyca‐forest‐gene‐bank‐collects‐dna‐of‐endangered‐plants‐in‐the‐bialowieza‐forest) gathers samples from the eastern European Białowieża Forest, The Pacific Center for Molecular Biodiversity (PCMB) (https://www.bishopmuseum.org/pcmb/) focuses on a concrete bioclimatic region, while Kew DNA Bank (https://dnabank.science.kew.org/homepage.html) has a wider scope and accepts samples from all over the world (Campbell, Astrin, Brody, et al. 2018). In other cases, eDNA collections like the Chicago Botanical Garden's DNA Biorepository (https://www.chicagobotanic.org/) comprise several individuals from each taxa, making them adequate for phylogenetic and taxonomic purposes.

In the recent years, the relevance of physical vouchers for DNA‐based studies has been highlighted (e.g., Dick and Webb 2012; Culley 2013; Gostel et al. 2016; Buckner et al. 2021), leading to the emergence of new terms referring to their relationship with genetic samples (Pleijel et al. 2008; Funk et al. 2018) (Table 1) or to their interconnection with all their associated natural and derived information (Webster 2017; Lendemer et al. 2020). This theoretical framework posits that vouchers and genetic samples should ideally derive from the same individual—that is, hologenophore direct voucher or, slightly suboptimally, an isogenophore direct voucher (Table 1) (Pleijel et al. 2008; Funk et al. 2018). Moreover, it emphasizes the relevance of preserving not only the phenotype and genotype, but also the ecological context (Webster 2017; Funk et al. 2018; Lendemer et al. 2020). Thus, a better understanding of species and populations would be achieving by creating Extended Specimens (ESs) and Extended Specimens Networks (ESNs) through the linkage of all direct and derived samples (e.g., genetic samples or histologic samples) and their metadata (e.g., site of collection and DNA sequence or histologic images) (Webster 2017; Funk et al. 2018; Lendemer et al. 2020). PBBs internal functioning generate *de facto* a collection of ESs and ESNs (mainly hologenophore direct vouchers) preserved under controlled conditions and available for researchers, something that contributes to the reproducibility of results without taxa resampling (GGBN 2015; Funk et al. 2017; Campbell, Astrin, Brody, et al. 2018; CETAF 2020). Besides, PBBs can contribute to natural collections by promoting large expeditions in collaboration with specialists from museums and botanic gardens—reducing the costs of plant biodiversity DNA‐based studies—and to scientific studies by providing services to researchers unfamiliar with molecular techniques and by generating MOAs and SOPs protocols' rankings (Mattick et al. 1992; Palacio‐Mejía 2006; Hodkinson et al. 2007; Zimkus and Ford 2014a, 2014b; Spooner and Ruess 2014a, 2014b; Lear et al. 2018; Campbell, Astrin, Brody, et al. 2018; Abdaljaleel et al. 2019; SANBI 2020; CETAF 2020). The reliability and low costs of DNA‐rich tissues preservation in silica gel allows sparing morphological voucher destruction (Staats et al. 2011; Gaudeul and Rouhan 2013; González‐Toral et al. 2022) and represents a great opportunity to compare its basal decay with that of DNA extracts preserved under controlled conditions.

**TABLE 1:** Types of morphological voucher regarding its relation to the samples of molecular studies and their taxonomic value described by Pleijel et al. (2008) and by Funk et al. (2018).

Type of voucher and definition by Pleijel et al. (2008)	Type of voucher and definition by Funk et al. (2018)
**Hologenophore**: The molecular sample and the voucher specimen are the same individual: a portion is used for the molecular studies while the rest is the deposited as morphological voucher. This is the optimal voucher.	**Direct voucher**: The voucher specimen is the source of the genetic sample.
**Isogenophore**: The molecular sample and the voucher specimen are not exactly the same individual, however there is a clonal relationship between them. This is the second optimal voucher.	
**Progenophore**: The molecular sample and the voucher specimen are not the same individual, although they are related through an ascendent/descendent or a full sibling relationship.	**Indirect voucher**: The voucher specimen is not the source of the genetic sample. Nevertheless, the voucher specimen is consider to be representative of the source of the genetic material since: (1) it belongs to the same taxon, (2) has been identified by an expert, (3) was collected at the same site by the same collector.
**Paragenophore**: The molecular sample and the specimen voucher were collected at the same location simultaneously. They are two distinct individuals without any known familiar relationship, which belong to the same population.	
**Syngenophore**: The molecular sample and the voucher specimen voucher are two different individuals which were collected at different locations or at the same location at a different time. There is not any known familiar relationship. Weakest voucher.	

**FIGURE 2:**
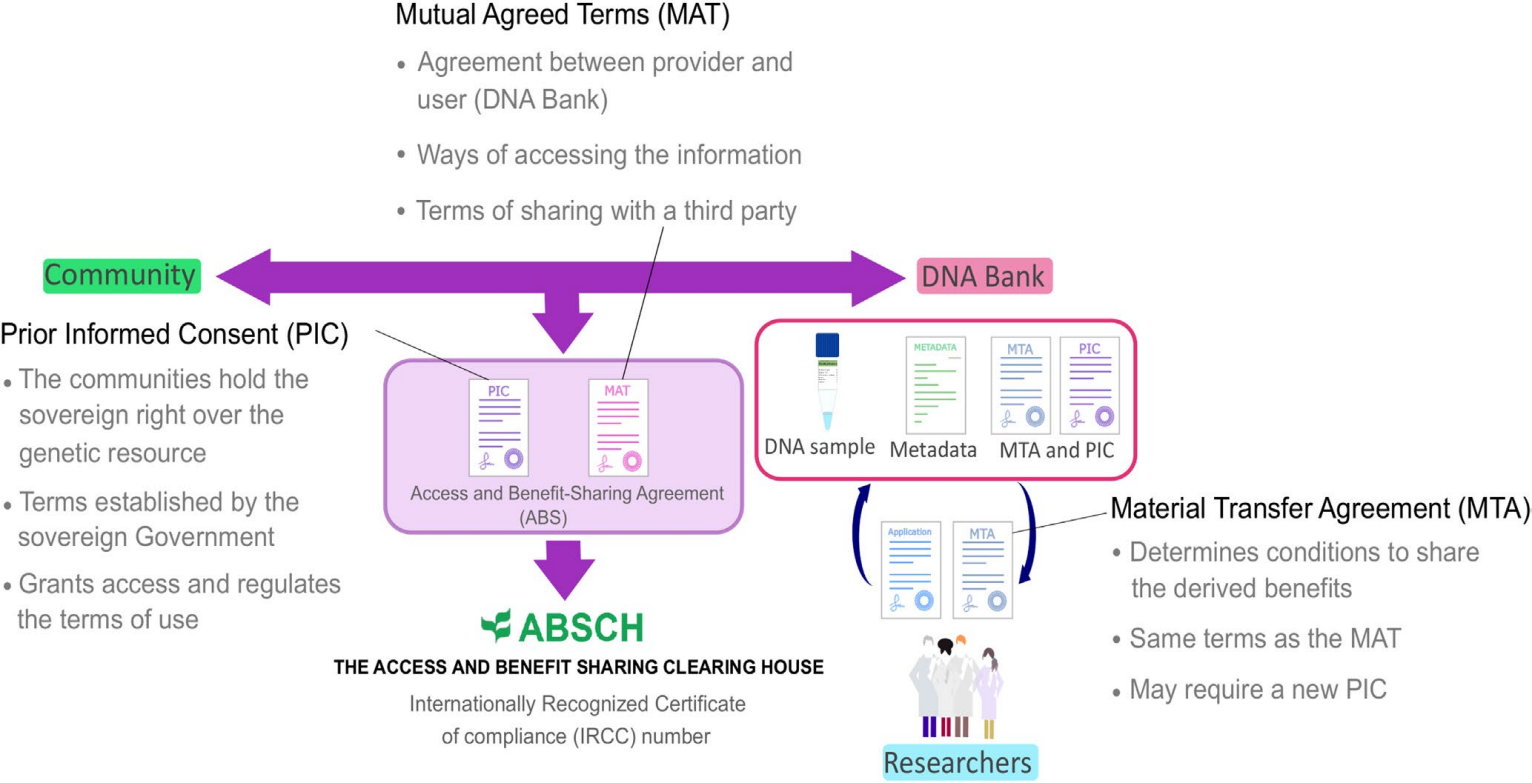
Scheme representing the different agreements required by the Nagoya Protocol for acquiring, donating, and loaning samples to a DNA bank while conveying with the international legislation.

The BDC prompted the development of regulatory international legislation for biodiversity, wildlife and genetic resources, including the Convention on International Trade in Endangered Species of Wild Fauna and Flora (CITES) (IUCN 1973), the International Treaty on Plant Genetic Resources for Food and Agriculture (ITPGRFA) (FAO 2001) and the Convention on Biological Diversity (CBD) and the Protocol of Nagoya (CBD 2011). Since expeditions, donations and samples exchanges are essential parts of biobanks' material collection and DNA utilization MOAs, PBBs activities make them play an important role in the implementation of these international legislations (Dröge et al. 2014; Zimkus and Ford 2014a, 2014b; Campbell, Astrin, De Souza, et al. 2018; CETAF 2020). The CBD and the Protocol of Nagoya (ratified by 126 countries) are the most relevant international legislations for PBBs, as they guarantee equality and justice by clarifying genetic resources' accessing and benefit‐sharing terms (CBD 2011; GGBN 2015; Biber‐Klemm et al. 2016; Davis and Borisenko 2017). The Protocol of Nagoya elaborated the CBD principles for the Access and Benefit‐Sharing (ABS) through Prior Informed Consents (PICs), which abides by the local legislation of the community holding the sovereign right over the sample and specifies the accessibility terms, and Mutually Agreed Terms (MATs), which unambiguously define the provider's monitoring process and usually include genetic material and traditional knowledge accessibility, publishing and storage policies (GGBN 2015; Biber‐Klemm et al. 2016; Davis and Borisenko 2017; Campbell, Astrin, Brody, et al. 2018). Once the PICs and MATs for the ABS are reached, their information is published in the Access and Benefit‐sharing Clearing‐House (https://absch.cbd.int/) by the provider's national competent authority to generate an Internationally Recognized Certificate of Compliance (IRCC) number, which should always be associated with samples (Davis and Borisenko 2017). The PICs and MATs terms have to be reflected in the biobank's Material Transfer Agreements (MTAs) when loaning samples in compliance with the CBD and the Protocol of Nagoya (Rice, Shepherd, et al. 2006; Zimkus and Ford 2014a; Biber‐Klemm et al. 2016; Davis and Borisenko 2017) (Figure 2). All ABS agreements require: (1) internal protocols for establishing relationships with the provider's competent authorities and implementing their policies, (2) defining technical approaches and the scope of scientific research, and (3) determining the benefit‐sharing terms, which are typically non‐commercial for PBBs (e.g., scientific training or future collaborations) (GGBN 2015; Davis and Borisenko 2017; Campbell, Astrin, Brody, et al. 2018; Campbell, Astrin, De Souza, et al. 2018; Williams 2019; CETAF 2020).

Therefore, PBBs are fundamental for generating and sharing tissue and DNA preservation SOPs, the creation of ESs and ESNs, and the implementation of national and international legislation for plant biodiversity preservation.

## 3 Changing the Scheme of PBBs Networks

The idea of creating a network to facilitate the connection between the existing biobanks emerged in the early days of DNA biobanking. The first initiative of this kind being DNA‐Bank Net, an ambitious project aiming to preserve endangered tropical plants' DNA across continents (Adams 1988, 1993; Spooner and Ruess 2014a). Initially, few scientists expressed interest. However, its scientific interest compelled scientists from 40 institutions across all continents by the early 1990s (Adams and Adams 1992; Adams 1993, 1997). This led to the 1991 meeting in which the nodes' structure was proposed, along with the idea of physically separating RN (or ideally the two RNs) and the WN by housing them in DNA banks facilities located on different continents (Adams and Adams 1992; Adams 1993, 1997; Graner et al. 2006; Spooner and Ruess 2014a). Despite its useful contributions and the 2000s estimations of the proportionally low costs of establishing DNA biobanks at a global scale, DNA‐Bank Net remained inactive by the mid‐2000s (Savolainen 2004; Graner et al. 2006; Hodkinson et al. 2007; Brown 2011; Spooner and Ruess 2014a). Furthermore, during these decades, public funding for taxonomic and systematics research declined to the point where the existing specimen collections, a legacy of generations of researchers, were threatened, despite their many social and scientific benefits.

Later on, the creation of a network of biodiversity biobanks was resumed by the decentralized pilot project DNA Bank Network, in which all types of biodiversity independent DNA collections coordinated to make their data accessible via the internet and generate a larger and more diverse pool of vouchers‐linked sequences (Gemeinholzer et al. 2011). In 2011, the DNA Bank Network evolved into the Global Genome Biodiversity Network (GGBN) an association that aims to standardize sharing procedures, ethical use and best practices while encouraging knowledge exchange and recruiting new members (Dröge et al. 2014; Seberg et al. 2016; GGBN 2024). The GGBN has been growing in the recent years: from 24 members in 2014 to 112 biorepositories from 38 countries, including at least 29 plant and fungi collections and 2 department collections (Dröge et al. 2014; GGBN 2024). A part from providing SOPs guides and ABS implementation models, GGBN's commitment with the Tree of Life led to the creation of the GGBN data portal (Dröge et al. 2014, 2016; Seberg et al. 2016). This portal addressed the problem of DNA banks samples information availability by allowing taxa and samples searches within the members' standing collections (having over 185,000 accessions from Kingdom Plantae) and by creating data standards (e.g., Sample PREanalytical Code (SPREC) or ABCD‐DNA) (Benson et al. 2010, 2011; Betsou et al. 2010; Dröge et al. 2014, 2016; Seberg et al. 2016; GGBN 2024). However, despite the GGBN globalization efforts, most members are not located within the highest plant biodiversity richness regions (Barthlott et al. 2005), but rather in western richer countries (GGBN 2024). On the other hand, there is no information on whether GGBN follows the RN relocation scheme of Adams (1997).

The Adams (1997) RN relocation scheme was based on the idea of preserving plant biodiversity by securing biobanks samples from all sorts of threats, including natural disasters. As one of the main aims of DNA‐Bank Net was to preserve as many species as possible at reduced costs, it focused on large collections (i.e., herbaria and botanic gardens) since their personnel is already familiarized with sampling SOPs (Adams and Adams 1992; Adams 1997). Although this was a well‐conceived scheme that addressed the main issues of plant biodiversity loss using large collections as backbone, it has not been implemented. Moreover, during the 1990s and 2000s, when the scientific community and the society were aware of the BDC, biodiversity collections faced funding reductions or even closures that have tremendously affected the scientific value of the remaining collections (Adams and Adams 1992; Dalton 2003; Lavoie 2013). Hence, the success of using large collections as backbone for preserving plant biodiversity depends, in part, on funding, the potential financial crises and political will. Since the Adams (1997) scheme has not been implemented worldwide (although there exist examples like the South African National Biodiversity Institute (SANBI) (SANBI 2020)), any disaster at any large collection would represent the loss of a large proportion of genetic information in a context were world biodiversity is on decline (Eichenberg et al. 2021).

It should also be highlighted that smaller DNA collections (i.e., Department collections) would be of interest for meeting the goal of preserving genetic material and metadata of as many species as possible. These collections have several appealing features to fulfill this task as they may (1) focus on a specific biogeographic unit and their related areas, and/or on specific groups of families, (2) present samples from species, subspecies or populations of conservational value (e.g., endemisms, relictic populations), (3) have associated morphological voucher material deposited in Index Herbariorum listed Herbaria, (4) many of the data derived from those samples has been published and is publicly available (e.g., Genbank sequences https://www.ncbi.nlm.nih.gov/genbank/), and (5) samples are usually preserved as tissues in silica gel and/or as DNA extracts in cold. For example, our DNA collection at the area of Botany of the department of Biology of Organisms and Systems of the University of Oviedo consists of DNA extracts preserved at −20°C and foliar tissue preserved in silica gel at room temperature. These samples comprises taxa and populations of taxonomic and conservational interest (e.g., Cires Rodríguez et al. 2012; Cires and Prieto 2015; González‐Toral et al. 2023) occurring in the Mountains Cantabrian and in the European Atlantic Biogeographic unit and others related to it (e.g., Mediterranean). The DNA collection samples are associated to morphological herbarium vouchers, most of them deposited in the Index Herbariorum listed Herbario de la Facultad de Ciencias de la Universidad de Oviedo (FCO) (Index Herbariorum 2023; Universidad de Oviedo 2023). These two ways of preserving DNA and DNA‐rich tissues represent an opportunity for following the Adams and Adams (1992) and Adams (1997) two‐node scheme from an early stage, as the WN could serve as the DNA extracts and the tissues preserved at room temperature could be used to build the RN. Furthermore, the MOAs could have a holistic approach focused not only on generating ESs (Webster 2017), but also on ESNs (Lendemer et al. 2020) by connecting the published data with the curated specimens (i.e., DNA and DNA‐rich samples with DNA sequences and herbarium vouchers).

Given their potential, smaller Department DNA collections like ours could play an important role in plant biodiversity conservation. By combining many of these collections, we can (1) cover wider floras and broader biogeographic regions than the larger existing collections, (2) cover lower taxonomic levels (e.g., subspecies, *forma*), and (3) focus on the genetic diversity of populations with local and/or regional conservational interest. Besides, given the critical funding dependence of large collections witnessed in the last decades, their susceptibility to catastrophes (e.g., fires, bombings, earthquakes…), and the chronical underfunding of plant conversation programs (Suárez and Tsutsui 2004; Balding and Williams 2016; Roberson and Meyer 2020; Tyler et al. 2023), the existence and maintenance of many smaller collections would secure samples survival. These smaller collections can be resilient in funding shortfall scenarios due to their low budget orientations. For instance, our foliar tissue collection at the University of Oviedo is preserved in silica gel at room temperature, and its maintaining costs are limited to the purchase of paper envelopes and reusable silica gel once in a wild. Furthermore, department collections like ours are built over decades through projects focused on the application of molecular techniques to plant taxonomy and conservation, and have been particularly active in generating scientific knowledge.

We believe that the current situation of PB biobanking is analogous to the 1940 Dunkirk evacuation (also known as Operation Dynamo). In May of 1940, 2 weeks after the beginning of the Battle of France (II World War) around 400,000 British, French and Belgian troops retreated to the French coastal village of Dunkirk after being trapped by the unstoppable advance of the German Army (Snyder 1960; Liddell Hart 1970). The British High Command decided to evacuate the Allied troops to Great Britain through the Channel, but this presented a problem: large ships would allow the evacuation of many soldiers at once at the expenses of becoming an easy target to the Luftwaffe (German Air forces) (Snyder 1960; Liddell Hart 1970). The British Admiralty came up with one idea: they would requisition all the possible “self‐propelled pleasure craft between 30' and 100' in length” for the evacuation, presenting the German Army with a plethora of smaller targets at once (Snyder 1960). The civilians of the Allied countries felt compelled by this call for vessels, forming a multinational eclectic flotilla of almost 900 vessels that managed to transport around 340,000 British, French and Belgian troops (Snyder 1960; Liddell Hart 1970). This operation was an unprecedented success, greatly surpassing the British Admiralty's initial estimate of rescuing 45,000 British troops (Liddell Hart 1970). Relying on a small number of larger collections as suggested by Adams (1997) ensures the preservation of many species at one place, but it also puts many samples at risk in the event of a catastrophic event, including financial ones. On the other hand, having a large number of smaller collections requires many more institutions involved and a higher level of coordination but, allows to preserve substantially more samples and puts at risk a smaller number of samples in case of catastrophe. Thus, this “Dynamo scheme” is based on the existence of many interconnected smaller collections that could all in all preserve more samples from taxa and populations. Therefore, although the current number of Department DNA collections is unknown, they may be of vital importance in future scenarios of Climate Change, species extinctions and population and species loss of genetic diversity.

## 4 Developing a Department PBB From Tissue and DNA Collections

**FIGURE 3:**
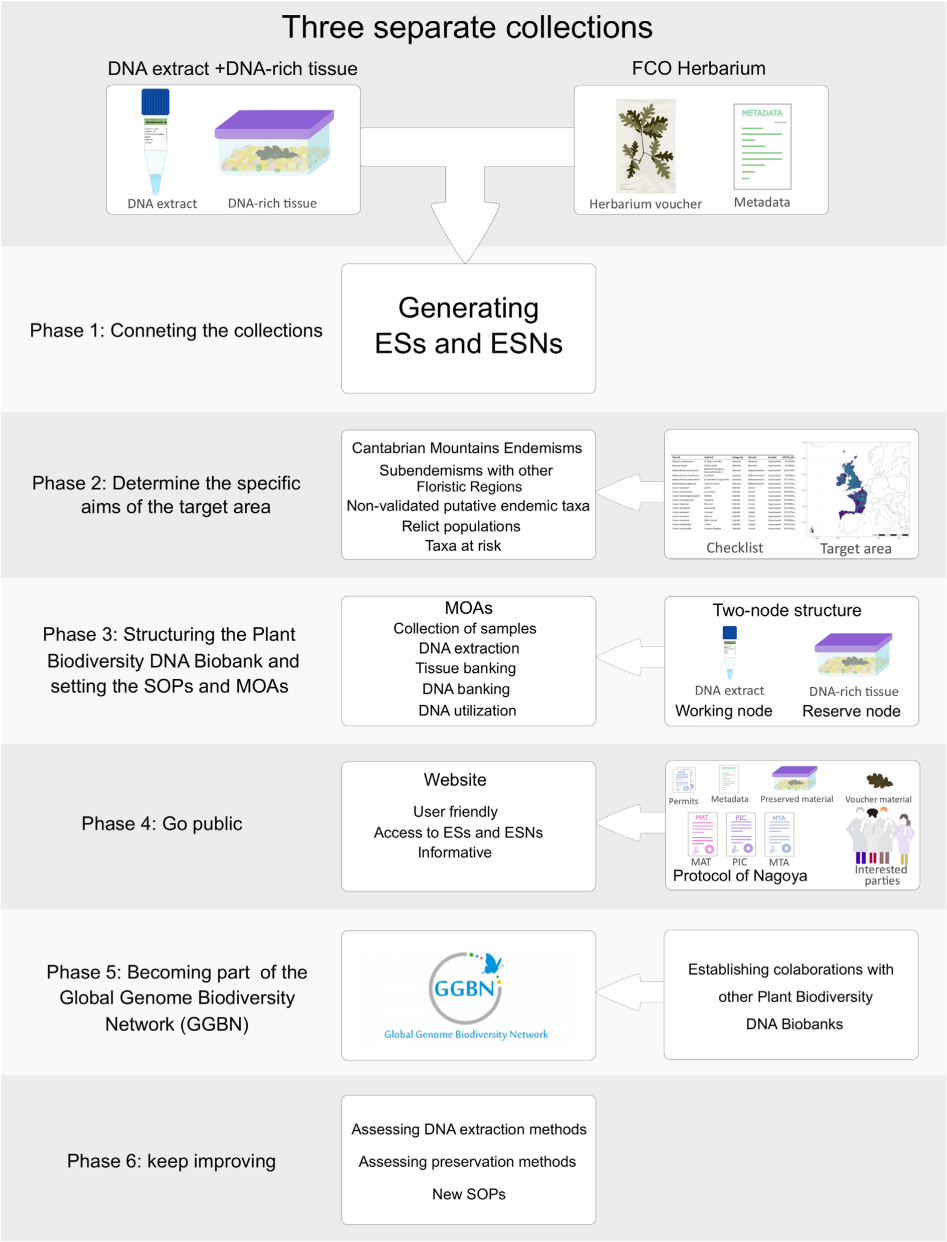
Scheme depicting the different phases of the process for transforming three separate collections into a Department Plant Biodiversity DNA biobank using the collections of the area of Botany of the department of Biology of Organisms and Systems of the University of Oviedo as example.

Given all the features and potential of department collections, we present a comprehensive 6‐step process for creating a small PBB focused on a specific floristic area and on generating ESs and ESNs (Figure 3). The steps on the existence of 3 separate department collections (hereafter the 3 collections): (1) the voucher material (e.g., herbarium vouchers), (2) the DNA‐rich tissue, and (3) DNA extracts collections. However, activities of these steps can be adapted to the number and type of collections and their associated MOAs. These phases aim to establish the PBB's structure and functioning, and to gather and assess the current knowledge of its target floristic area, and catalog specimens as we make them available. As we pursue a holistic approach that facilitates information sharing and the Dynamo scheme, collaboration with existing PBBs and digitalizing and centralizing specimen open‐access information will be fundamental to this process.

### 4.1 Phase 1: Connecting DNA Collections to Herbarium Vouchers and Metadata: Creating an Extended Specimens (ESs) Collection

Initially, the DNA collection specimens (e.g., the DNA extract and/or the DNA‐rich tissue samples) should be linked to their herbarium vouchers and metadata, any publicly available genetic information (e.g., Genbank ID number) and the scientific studies from which they originate. This is crucial for creating ESs and ESNs and involves creating an inventory of published information associated with each sample. The PBB foundations would be a private database connecting the three separate collections (or two collections if there is no tissue collection) and the physical connection of their specimens via ID numbers and barcodes or QR codes. Metadata deficiencies detected during this process would improve the data gathering process, impacting positively on SOPs, ESs, ESNs and FAIR data and implementing the Responsible Research and Innovation (RRI) principles (Hodkinson et al. 2007; Owen et al. 2012; Funk et al. 2017; Williams et al. 2020).

Three fundamental aspects are relevant during this phase: (1) taxa names update and standardization, (2) metadata gathering and standardization, and (3) sampling strategy. The first aspect could be solved by following the criteria of World Checklist of Vascular Plants (WCVP) and Plants of the World Online (POWO) (Govaerts et al. 2021; Govaerts 2023; Royal Botanic Gardens Kew 2025) during an initial state and then modifying it according to the latest literature. The DNA collection's specimens database should ideally include: (1) taxon name, (2) taxonomy (i.e., kingdom, division, class, order), (3) accepted genus (including author(s)), (3) species accepted (including author(s)), (4) infraspecific accepted taxon, if applicable, (5) accepted species WCPV number and its URL, (6) location (including coordinates and altitude), (7) collection date, (8) collector(s), (9) voucher ID number, (10) DNA extract and/or tissue sample ID, (11) type of study conducted on samples (i.e., phylogenetic vs. genetic diversity), (12) molecular information obtained from the sample (i.e., sequenced markers or genomic sequencing), (13) sample's National Center for Biotechnology Information (NCBI) ID (i.e., GenBank number), if applicable and (14) publication (e.g., Title, DOI) (see Table S1 and Table S2 for more detail). Future acquisitions would benefit from the inclusion ecological data during the collection process, including generating photovouchers, which can be easily obtained with cell phones. Special attention should be given to the recommendation of Gemeinholzer et al. (2010), Davis (2011), Culley (2013), Gostel et al. (2016) and Funk et al. (2017) regarding recording the presence of water streams, the soil type or the habitat (Table 2). We classified sampling strategies found in the University of Oviedo collection can be classified into three categories: (1) taxonomic sampling, consisting in one or few individuals from various taxa sometimes including type locations (e.g., *Rivasmartinezia vazquezii* Fern.Prieto & Cires (2014), *Micranthes stellaris* (L.) Galasso, Banfi and Soldano (2005)), (2) genetic diversity sampling, comprising several individuals from the same taxa belonging to various populations (e.g., *Cochlearia pyrenaica* DC. (1821), *Ranunculus cabrerensis* Rothm. (1934)) and (3) distribution validation sampling, consisting in numerous individuals from one or more taxa throughout a wide geographical range (e.g., *Cytisus dieckii* (Lange) Fern.Prieto, Nava, Fern.Casado, M.Herrera, Bueno Sánchez, Sanna and Cires (2017), *Ulex cantabricus* Álv.Mart., Fern.Casado, Fern.Prieto, Nava and Vera (1988)). Hence, some taxa are represented by few individuals, while others count with dozens of individuals. Moreover, samples collected following a genetic diversity or a distribution validation sampling schemes are more likely to have associated ecological data than those obtained during taxonomic sampling. Besides, genetic diversity studies raw results have often not been made publicly available, with only genetic parameters or statistical genetic analyses being published, highlighting the need for a method to publish these raw data and facilitate the generation of ESs and ESNs.

**TABLE 2:** Modifications of the main categories of collection data proposed by previous authors and additions to the specific information.

Data category	Specific data
***Collectors***	(1) Collector's full name, (2) Collector's affiliation, (3) Collection site, (4) Collection number (= collector surname(s) and a number unique collector's number), (5) Collecting date, (6) Collecting expedition and/or Project, (7) Data of other members of the collecting expedition (Prendini et al. 2002; Davis 2011; Culley 2013; Funk et al. 2017; Lear et al. 2018; Martin et al. 2019).
***Sampling***	(8) Collection procedures: **in situ *DNA extraction/Drying/Freezing/In solution*** (9) Detailed preservation treatments, ***(10) Sample type: DNA/DNA‐rich tissue, (11)Sampling scheme: Taxonomic sampling/Genetic diversity sampling/Distribution validation sampling*** (Prendini et al. 2002; Davis 2011; Culley 2013; Funk et al. 2017; Lear et al. 2018; Martin et al. 2019).
Biological	(12) Scientific name of the taxon, (13) local popular name, (14) plant description: (14.1) Life stage, (14.2) Habit, ***(14.3) Monoic/Dioic: masculine/feminine***, ***(14.4) Flower presence/Fruit presence*/**Spores presence, ***(15) Life span: annual/biannual: first year/s year/other*, (*16) Raunkiær (1934) Plant life‐from: Phanerophyte/Epiphyte/Chamaephyte/Hemicryptophyte/Cryptophyte/Therophyte/Aerophyte, (17) Further Raunkiær (1934) subdivisions*** (Davis 2011; Gostel et al. 2016; Funk et al. 2017).
Geographical	(18) Location name, (19) County and/or other political administrative divisions, (21) Country, (22) GPS coordinates (23) Description of the sampling site: (23.1) Distance to the nearest or the presence of nearby streams, ***(23.2) Distance to the nearest road***, ***(23.3) Directions to reach the location***, **(*23.4) Type of area: wild/rural/city/suburbia***, ***(23.5) Observations***, (24) Altitude (Gemeinholzer et al. 2010; Davis 2011; Culley 2013; Funk et al. 2017).
Ecological	(25) Habitat, ***(26) EUNIS habitat code***, ***(27) Dominant species in the area***, (28) Surrounding species, ***(29) Photovoucher***, (27) Soil type, ***(30) Humidity***, ***(31) Temperature***, ***(32) Floristic Province***, ***(33) Floristic Subprovince, (34) Presence of alien invasive species: Yes/No, (35) Alien invasive species*** (Davis 2011; Culley 2013; Funk et al. 2017).
***Distributional***	***(36) Distribution type: Cosmopolitan/European/Subendemic/Endemic, (37) Type location: Yes/No, (38) Location within range: typical/marginal (including isolated populations)/controversial/new.***
***Conservational***	***(39) IUCN status: NE/DD/LC/NT/VU/EN/CR, (40) International legal regulation(s), (41) Local status: NE/DD/LC/NT/VU/EN/CR, (42)Local legal regulation(s), (37) Collecting permissions.***

*Note:* These additions aim to enhance resampling capacity and address potential needs of the identified sampling types in our University of Oviedo collection DNA collection: taxonomic sampling, genetic diversity sampling, and distribution validation sampling. The new additions and/or modifications are highlighted in italics and bold lettering.

### 4.2 Phase 2: Determining the Specific Aims and the Target Area of the PBB

The establishment of target areas and conservational goals must be a keystone for PBBs given the current conservational needs and the biodiversity loss (De Vos et al. 2015; Nic Lughadha et al. 2020). Consequently, efforts should focus on comprehensively cataloging the flora of the target area to establish conservational and scientific priorities. We classified these taxonomic, phylogenetic, and conservational priorities in six groups: (1) target area endemics, (2) subendemics shared with other floristic regions, (3) putative endemic taxa with non‐molecularly validated taxonomic status, (4) relictic and/or isolated populations, (5) taxa at global conservation risk, and (6) taxa at local conservation risk. Furthermore, a detailed inventory could unveil understudied and/or undersampled taxa and areas, which will be relevant for future conservation strategies in collaboration with other biobanks.

Alien Invasive Species (AIS) are capable of causing a negative impact on biodiversity, socioeconomic activities and human health during their establishment and dissemination (Kolar and Lodge 2001; Keller et al. 2011; Ricciardi 2013). AIS pose the second most important threat to biodiversity due to their intimate relation with human activities, such as international commerce, and their ability to modify the environment (e.g., nutrient cycles or soil features) (Westphal et al. 2008; CBD 2000; Vilà et al. 2011; Ricciardi 2013; Castro‐Díez et al. 2019; Shabani et al. 2020). The collection of AIS genetic material within invaded areas will be of aid in understanding biological invasions features, including: (1) the number of events of introduction, (2) potential origins of colonization waves, (3) temporal and spatial invasion dynamics, (4) predominant reproduction type (i.e., vegetative vs. sexual), and (5) genetic diversity difference between their invasive and natural ranges. Consequently, establishing AIS sampling as one of PBBs conservation priorities will enhance our understanding of AIS spread and improve monitoring strategies.

### 4.3 Phase 3: PBB Structuring and Setting the Standardized Operating Procedures (SOPs) of the Major Operational Activities (MOAs)

**FIGURE 4:**
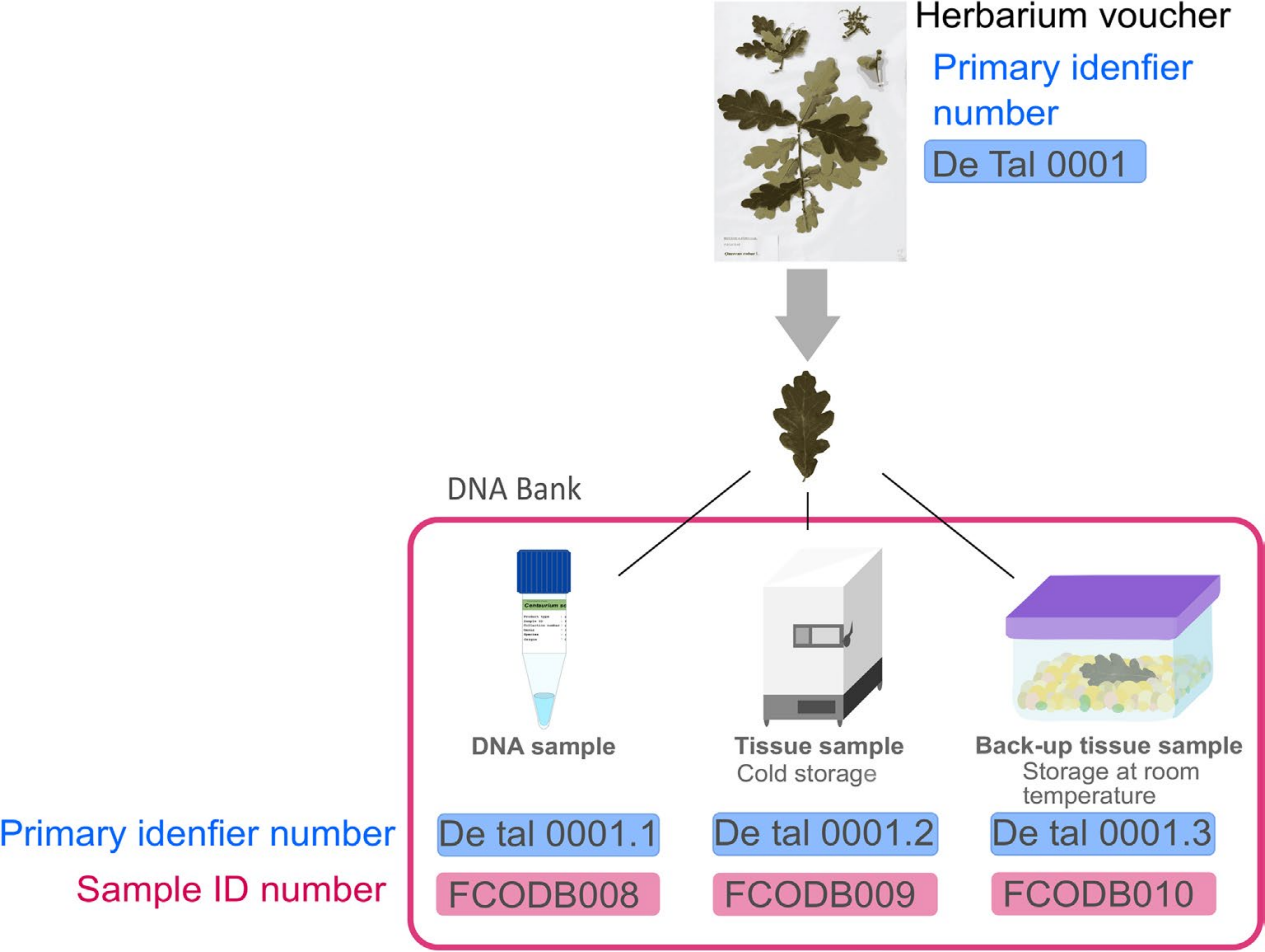
Scheme depicting an example of the Funk et al. (2017) double identifying number scheme. The collecting number of a voucher used as primary identifier number of all the derived vouchers and the sample ID numbers of all tissues in DNA bank storage derived from the same sample. The collection number or primary identifier number connects the voucher and the samples since it is formed by the collecting number (in this case De Tal 00001) plus an additional number (e.g., De Tal 00001.1). When acquired by the DNA bank, another unique internal number, the sample ID number, analog to the Herbarium catalog number, is given to each of sample (in this case FCOD008).

DNA extracts and DNA‐rich tissue collections can be effectively organized into the recommended two‐node PBB using inventories: DNA extracts collection would constitute the WN, and the tissue collection would form the RN (Adams and Adams 1992; Mattick et al. 1992; Adams 1997; Rice, Henry, and Rossetto 2006; Hodkinson et al. 2007; Zimkus and Ford 2014a, 2014b; Campbell, Astrin, Brody, et al. 2018). DNA and tissue banking would be the first MOAs *sensu* Hodkinson et al. (2007) to have their SOPs established. Reasonable high‐quality DNA preservation can be achieved at low costs by storing DNA extracts at −20°C and DNA‐rich tissues in silica gel at room temperature (Prendini et al. 2002; Corthals and De Salle 2005; Hodkinson et al. 2007; Zimkus and Ford 2014a, 2014b). Building the ESs collection would require high levels of traceability, which could be achieved by implementing the Funk et al. (2017) double identifying number scheme. In this scheme each sample has two identifying numbers: a primary identifying number derived from the voucher collecting number that links ES samples and a second DNA Bank collection identifying number that is unique to each sample (Figure 4) (Caujapé‐Castells et al. 2010; Funk et al. 2017; Campbell, Astrin, Brody, et al. 2018). At this early stage, the labelling system should also follow the recommendations for loaning samples from international organizations (e.g., World Health Organization (WHO) 2003; Campbell, Astrin, Brody, et al. 2018; Instituto de Investigación de Recursos Biológicos Alexander von Humboldt (IPT IAvH) 2020).

**FIGURE 5:**
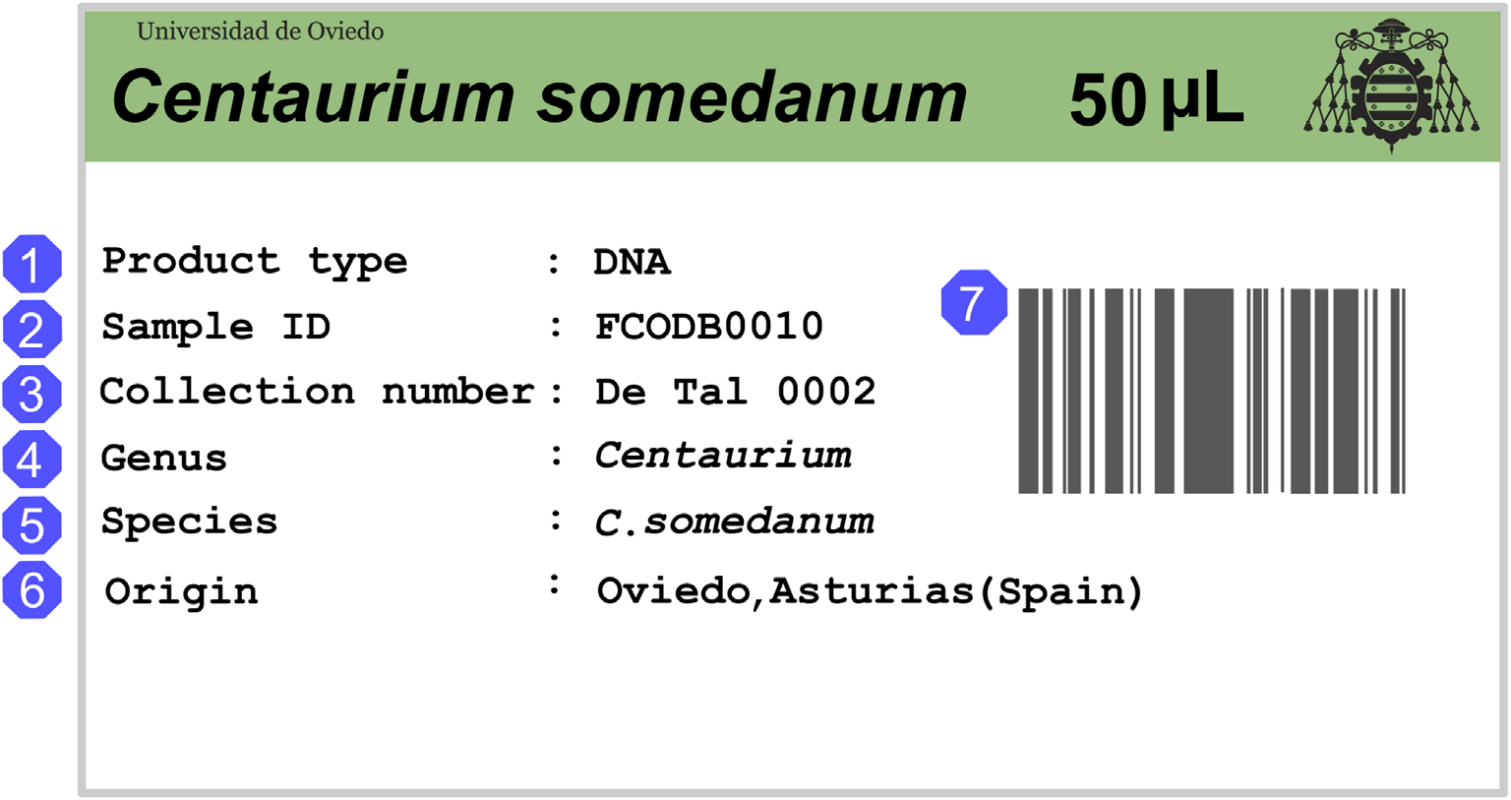
Example of potential label of loaned samples following the instructions of World Health Organization (WHO) (2003), Caujapé‐Castells et al. (2010), Campbell, Astrin, Brody, et al. (2018), and Instituto de Investigación de Recursos Biológicos Alexander von Humboldt (IPT IAvH) (2020). These authors believe that the label of a loaned sample should specify: The type of product (DNA, RNA or tissue) (1), the sample ID number of the biobank (2), the collection number (3), the genus of the sample (4), the species of the sample (5), the origin of the sample (6) and a barcode (7).

A preliminary label design for the ESs collection should include: (1) product type (DNA, RNA or tissue), (2) collection number (= primary identifying number), (3) biobank ID number, (4) accepted genus, (5) accepted species, (6) sample origin, and (7) an assigned barcode or QR code (WHO 2003; Caujapé‐Castells et al. 2010; Campbell, Astrin, De Souza, et al. 2018; IPT IAvH 2020) (Figure 5). At this stage the collection would comprise samples obtained before and after the centralization process started. We suggest that three different labels should be used: (1) the labels of samples collected before the centralization (i.e., initial ESs), (2) the collection labels (i.e., samples acquired after the SOPs were set), and (3) the loan labels. During this phase, the collection would comprise samples obtained before and after the centralization process started. We suggest that three different labels should be used: (1) the labels for samples collected before the centralization (i.e., initial ESs), (2) the collection labels for samples acquired after the SOPs were established, and (3) the loan labels. These three label types should be easily recognized (e.g., by using different colors or patterns) to guarantee sample's traceability.

Establishing the aims and priorities and the interconnection between the three collections, phases 1 and 2, would benefit material collection, data gathering and sample labelling SOPs by highlighting new ways of improving ESs and ESNs to match the scientific necessities. For example, updating the Flora checklist brings the opportunity to centralize ecological (e.g., European Nature Information System (EUNIS) habitats (https://eunis.eea.europa.eu/), IUCN Habitats Classification Scheme (https://www.iucnredlist.org/resources/habitat‐classification‐scheme), or floristic subprovince), distributional (e.g., endemisms, subendemisms or isolated populations) and conservational data (e.g., IUCN (https://iucn.org/) assessments or local assessments); while implementing local legislation and the Protocol of Nagoya. We strongly believe that all this information should be part of the SOPs related to ESs and ESNs. Consequently, we propose combining and extending the data collection items suggested by previous authors (e.g., Prendini et al. 2002; Culley 2013; Lear et al. 2018) to specify the following details: (1) national and international conservation status, (2) sampling location features regarding distribution range, (3) the sampling scheme, and (4) habitat features and classification (Table S1).

At a second stage, sample collection and DNA extraction and utilization SOPs could be established, implementing legal documents for donations, collections and loans while abiding regional, national and international legislations (e.g., the Protocol of Nagoya) (Davis and Borisenko 2017; CETAF 2020). This will enforce the ABS agreements by establishing the guidelines to generate PICs, MATs and MTAs, which are essential to sample collection and DNA utilization (CBD 2011; GGBN 2015; Biber‐Klemm et al. 2016; Davis and Borisenko 2017). International organizations like the Middle Eastern and African Society for Biopreservation and Biobanking (ESSB) (https://esbb.org/), the Consortium of European Taxonomic Facilities (CETAF) (https://cetaf.org/) or the International Society for Biological and Environmental Repositories (ISBER) (https://www.isber.org/) can facilitate this thanks to their remarkable contributions to DNA biobanking like producing documents for the ABS agreements implementation and particularly MTAs for DNA samples (e.g., GGBN 2015; CETAF 2020) or the ISBER Best Practices Guidelines (Campbell, Astrin, Brody, et al. 2018). The department PBB's transparency and accountability would be further strengthened by establishing a governance structure during this phase. The department PBB collection manager and the contact person(s) for sample providers and requesters, along with their responsibilities, should be clearly specified.

### 4.4 Phase 4: Go Public: From Three Collections to Extended Specimens (ESs) and Extended Specimens Networks (ESNs)

Once the SOPs are established and the three collections are integrated, a website should be developed with the following characteristics: (1) be user‐friendly, (2) interconnect specimens' data generating ESs and ESNs, and (3) be informative regarding the biobank functioning, samples' availability and the donation/request process. The best practice guides and ABS agreements guidelines generated during phase 4 would be of capital importance for the informative and user‐friendly aspects. Furthermore, generating ESs and ESNs from herbarium vouchers would upgrade the ongoing herbarium digitalization processes by producing and fomenting FAIR data and the RRI (Owen et al. 2012; Wilkinson et al. 2016; Lannom et al. 2019). Opening the website to the public would be the final step of the three collections interconnection.

### 4.5 Phase 5: Becoming Part of Global Genome Biodiversity Network (GGBN) and Other Organizations and Establishing Collaborations

Once the PBB is functioning, becoming part of the GGBN should be the next step, as the GGBN open to recruiting new members and aims to standardize the MOAs (Dröge et al. 2014; Seberg et al. 2016; GGBN, 2024). This membership would improve the PBB visibility among researchers and encourage collaborations other biorepositories. During this phase, the PBB could also aim to become part of other associations like ESSB (https://esbb.org/), CETAF (https://cetaf.org/) or ISBER (https://www.isber.org/).

The early idea of relocating the RN in other countries or harboring samples from other biobanks (Adams 1997; Graner et al. 2006; SANBI 2020) and has been implemented by some PBBs like the SANBI DNA bank (https://www.sanbi.org/biodiversity/foundations/genetics‐services/) and the Kew DNA Bank (https://dnabank.science.kew.org/homepage.html). While other PBBs are willing to do so (e.g., Jardín Botánico Canario “Viera y Clavijo” (http://www.jardincanario.org/banco‐de‐adn‐motivacion)). In this sense, sharing and exchanging samples would benefit both biobanks, especially when both share floristic elements. For instance, two different PBBs could harbor samples of closely related taxa occurring in different bioclimatic regions or of relictic and non‐relictic populations or samples from non‐native taxa at locations where they behave as AISs and at locations where they are innocuous species. In this way, active interactions and partnerships with other biobanks have the potential of boosting research in fields like biogeography, conservation biology or taxonomy.

### 4.6 Phase 6: Keep Improving

Since high‐quality DNA preservation is a key aspect of biobanking, the PBB should focus some of its research activity on finding and assessing new preservation methods to lower costs and risks. The performance of different solutions allowing storing DNA at room temperature (Wan et al. 2010; Clermont et al. 2014) or DNA extraction methods applied to different plant groups could be assessed, making methodological contributions to the scientific community. Similarly, finding new ways of improving ESs and ESNs should be an aim of PBBs.

### 4.7 Adaptations for Limited Funding or Infrastructure

The six‐step process can be adapted to limited funding or infrastructure by building a collection based on DNA‐rich tissue samples in silica gel at room temperature. Fortunately, the SOPs of this type of preservation can be accomplish on a low budget and with little previous experience in molecular research. The department PBB would manage the RN and its MOAs in their facilities, focusing mainly on sample collection and tissue banking. The MOAs must also be adapted to the fact that when the department PBB becomes active, WN (i.e., DNA extracts) would we outsourced to other facilities, specifying how and by whom the DNA extract will be managed.

Phase 4 involves making the department PBBs data publicly available through the internet. Although developing a website would be ideal, public repositories like EU Open Research Repository {Zenodo} (https://zenodo.org/) or Dryad (https://datadryad.org/) can serve this purpose. The relevant documentation (e.g., department PBBs structure and functioning, aims and target area…) can be uploaded with the appropriate copyright, while the user‐friendly experience can be achieved through clear display and linking of the information. Sample consultation could be facilitated through files in easily readable formats such as Comma Separated Values (CSV) or Excel (XLSX). These files should include (1) sample's ID in the RN and in external WNs, (2) level of access restriction of the sample's information, (3) accepted taxa name and other taxonomic data, (4) country where it was sampled, (5) contact information of the managers of the WN and RN where the specimens are deposited, (6) MOAs and SOPs documents that should be read, and (7) necessary hyperlinks and citations to generate ES and ESN.

The improvement of the department PBB preservation techniques could be achieved in collaboration with the institutions hosting the DNA extracts of the external WN(s).

## 5 Discussion

The BDC—one of the main ecosystemic, societal and economic threats to humanity—is characterized by extinction rates 1000 times higher than expected and has been exacerbated during the past decades by factors such as Climate Change, habitat loss and fragmentation or AIS, leading to predictions of reaching 10,000 times the background extinction rate (Feeley and Silman 2010; Dullinger et al. 2012; De Vos et al. 2015; Ceballos et al. 2017; Muthukrishnan and Larkin 2020; Shabani et al. 2020; Bellard et al. 2021; Eichenberg et al. 2021; Prakash and Verma 2022; Pörtner et al. 2023; Williams et al. 2023). The scientific community addressed this threat by generating and making available new phylogenetic knowledge, while national administrations and international organizations set different aims and funded biodiversity conservation programs like the Global Strategy for Plant Conservation (GSPC) of the United Nations (Blackmore 2002; United Nations (UN) 2021). Despite these efforts, plant species decline and extinctions continue, which can have unpredictable and disproportioned effect on ecosystems (Grime 2002; Schleuning et al. 2016; Eichenberg et al. 2021). More than 170 plant species have gone extinct in the wild since the 1960s, around 50,000–75,000 may currently be at risk of extinction and many could go extinct before even been described (Cronk 2016; Pimm and Raven 2017; Willis 2017; Ruta et al. 2020; IUCN 2024). Moreover, the prevention of local and global plant extinctions faces various additional problems: (1) chronic lack of funding (Suárez and Tsutsui 2004; Balding and Williams 2016; Roberson and Meyer 2020), (2) lack of time for sampling the declining species and populations due to the synergistic interactions between extinction‐driving factors (e.g., climate change and AIS), (4) absence of unified species conservation and AIS eradication strategies sometimes even within the same country (e.g., *Acacia mearnsii* De Wild. (1925)), *Cortaderia selloana* (Schult. & Schult.f. Asch. and Graebn. (1900)), (5) inefficient or incomplete systems of information sharing (i.e., there is not a conservation strategies database that facilitates decision‐making based on previous experiences), and (6) the reduced percentage of species with genetic information (Nic Lughadha et al. 2020).

Species and populations genetic data are as relevant for plant species conservation as habitat restauration and landscape dispersal securing (Aavik and Helm 2018). Centralizing and systematizing Department DNA collections would be of aid in implementing the genetic information use in conservation programs and would improve IUCN assessments' accuracy (Garner et al. 2020). Furthermore, the Dynamo scheme, by connecting smaller collections, could boost collaborations in genetic diversity studies and conservation efforts as different collections could contribute by sampling local populations, thus covering a larger proportion of the distribution range. Moreover, prioritarian taxa inventories would encourage conservational information sharing, including AIS dynamics and eradication strategies. In this context, more efficient conservation plans and the ABS agreements implementation (CBD 2011) would also benefit society by preserving and regulating the plant genetic and biodiversity heritage while addressing the GSPC goals (UN 2021).

During the last decade, initiatives of international organizations like the GGBN's Global Genome Initiative‐Gardens (GGI‐Gardens) (https://naturalhistory.si.edu/research/global‐genome‐initiative), the Biodiversity Collections Network (BCoN) (https://bcon.aibs.org/) or the Distributed System of Scientific Collections (DiSSCO) (https://www.dissco.eu/what‐is‐dissco/the‐collections) have contributed to natural collections digitalization and to completion of the vascular plants genetic barcoding (Gostel et al. 2016; Zúñiga et al. 2017). In the actual context, generating ESs collections and ESNs databases and providing updated inventories at a global scale could be viewed as the next logical step to these digitalization initiatives. Nevertheless, the proposed initial steps to this process, like creating a common database similar to the Index Herbariorum (http://sweetgum.nybg.org/science/ih/) listing the existing PBBs, have not been implemented at a global scale (Spooner and Ruess 2014a), although the USA achieved this through the Integrated Digitized Biocollections (https://www.idigbio.org/).

Our six‐step plan for transforming three separate collections into a ESs and ESNs PBB would benefit several scientific fields—for example, molecular taxonomy, conservational biology, DNA barcoding or eDNA—thanks to its positive impact on research results reproducibility and reliability (Corthals and De Salle 2005; Rice, Henry, and Rossetto 2006; Astrin et al. 2013; Spooner and Ruess 2014b; De Vere et al. 2016; Lear et al. 2018). This process represents additional advantages for local and regional conservational and research goals. For instance, our collection focuses on the Cantabrian Mountains flora (northern Iberian Peninsula), establishing a small PBB would imply the application of FAIR data and RRI principals and achieving some the goals for research improvement and sustainability of European Union's (EU) Horizon Europe (Owen et al. 2012; Wilkinson et al. 2016; Lannom et al. 2019; European Union (EU) 2021), while giving a new dimension to the EU initiative Innovation and Consolidation for large‐scale Digitisation of natural heritage (ICEDIG) (European Commission 2023) by generating ESNs (e.g., connecting herbarium voucher occurrences with their genetic data). Moreover, PBBs would make the goals of international barcoding projects such as the International Barcode of Life Project (iBOL) (https://ibol.org/), Encyclopedia of Life (EOL) (https://eol.org/) or The Barcode of Life Data System (BOLD) (https://www.boldsystems.org/) easier to achieve, while other fields like eDNA, Single Nucleotide Polymorphisms (SNPs) genotyping, transcriptomics or phylogenomics would further enhance the accuracy of these projects (Adams 1997; Rice, Shepherd, et al. 2006; Hanner and Gregory 2007; Pleijel et al. 2008; Rice et al. 2008; Astrin et al. 2013; Spooner and Ruess 2014b; De Vere et al. 2016).

The Dynamo scheme proposes rebalancing the current global PBB strategy to get the resilient smaller collections more involved in building PBBs networks. Networks robustness would benefit from the inclusion of department collections, which are likely to establish new connections and collaborations with research groups that curate complementary collections and share similar scientific interests. However, to capitalize on this potential, department collections need frameworks for developing small PBBs adapted to their own idiosyncrasies and based on the structure and functioning of the large institutions. Our six‐step process was designed to improve our knowledge on the relatively unstudied local Cantabrian Mountains vascular flora through the generation of a database and an updated inventory (Izco 1980; Fernández Prieto and Vázquez 2007; Fernández Prieto et al. 2014; Gómez Durán 2014), aiming to unveil taxonomic knowledge gaps and the factors associated to local biodiversity hotspots. With this information, the promotion of large expeditions focused on understudied areas and taxonomic groups would simultaneously contribute to the Leipzig Catalog of Vascular Plants and tackle one of the major bottlenecks for genetics‐based studies (Mattick et al. 1992; Lear et al. 2018; SANBI 2020; Freiberg et al. 2020). Particularly, ecology and molecular taxonomy would potentially profit from an updated species catalog thanks to data accuracy refinement and the possibility of sampling the type localities of taxa with scientific interest. In this sense, University of Oviedo department collections holds a very good example of the relevance of implementing DNA sampling when describing new taxa. Our collections includes specimens from the only two known species of the subendemic genus *Rivasmartinezia* Fern.Prieto and Cires (2014)—*Rivasmartinezia vazquezii* Fern.Prieto and Cires (2014) and *Rivasmartinezia cazorlana* Blanca, Cueto, Benavente & J.Fuentes (2016)—collected from their type localities (Fernández Prieto and Cires 2013; Blanca et al. 2016). Consequently, we could provide new molecular information (e.g., sequencing new different molecular markers) to the already available data at GenBank extending de facto ESs and ESNs without resampling. In this way, many small department PBBs connected through the Dynamo scheme could enhance information sharing by generating and expanding ESs and ESNs, even when they are type specimens. Furthermore, international digitalization and biodiversity projects would progress more rapidly in our biodiversity loss contexts if there existed many small department PBBs able to provide this type of specimens following standardized protocols.

## Author Contributions

**Claudia González‐Toral:** conceptualization (equal), funding acquisition (supporting), methodology (equal), writing – original draft (lead), writing – review and editing (supporting). **Eduardo Cires:** conceptualization (equal), funding acquisition (lead), project administration (lead), supervision (lead), writing – original draft (supporting), writing – review and editing (equal).

## Funding

This work was supported by Ramón Areces Foundation and the 2002166‐ Programa Severo Ochoa of the Government of the Principality of Asturias (XXXVI Convocatoria para Ampliación de Estudios en Gobierno del Principado de Asturias, 2002166‐ Programa Severo Ochoa).

## Conflicts of Interest

The authors declare no conflicts of interest.

## Supporting information


**Table S1:** Summarized example of the data gathered from each of the DNA extracts and/or tissue samples of the collections of the area of Botany of the department of Biology of Organisms and Systems of the University of Oviedo. PhG refers to phylogenetic studies; GD refers to Genetic Diversity studies, WCVP refers to the World Checklist of Vascular Plants by Royal Kew Gardens, Mol. Data refers to Molecular data, that is, the amplified sequence or the type of molecular marker studied in the published study that used the samples, NCBI ID refers to (GenBank) identifying number.

**Table S2:** Summary of the ideal metadata gathered to generate an Extended Specimens (ESs) and Extended Specimen Networks (ESNs). Ideally, this data should be added to the collection data (see Table 3).

## Data Availability

The data supporting this viewpoint are available within the article.
