# Supplementary material for: Extending Specimens to Save Plant DNA: Structuring Department DNA Collections in Times of Biodiversity Loss
Source: Ecol Evol. 2026 Apr 8;16(4):e73211. doi: 10.1002/ece3.73211 (PMC13059098; doi:10.1002/ece3.73211)
Supplement: Supplementary file 1 — Table S1: Summarized example of the data gathered from each of the DNA extracts and/or tissue samples of the collections of the area of Botany of the department of Biology of Organisms and Systems of the University of Oviedo. PhG refers to phylogenetic studies; GD refers to Genetic Diversity studies, WCVP refers to the World Checklist of Vascular Plants by Royal Kew Gardens, Mol. Data refers to Molecular data, that is, the amplified sequence or the type of molecular marker studied in the published study that used the samples, NCBI ID refers to (GenBank) identifying number. [file ECE3-16-e73211-s002.doc]

**Table S1**. Summarised example of the data gathered from each of the DNA extracts and/or tissue samples of the collections of the area of Botany of the department of Biology of Organisms and Systems of the University of Oviedo. PhG refers to phylogenetic studies; GD refers to Genetic Diversity studies, WCVP refers to the World Checklist of Vascular Plants by Royal Kew Gardens, Mol. Data refers to Molecular data, i.e. the amplified sequence or the type of molecular marker studied in the published study that used the samples, NCBI ID refers to (GenBank) identifying number.

| **Taxon** | **Class:Order** | | **Family** | | **Genus** | | **Species** | | **Species' Author & year** | | **Subsp./ Var./f. etc** | | **Author& year** | | **WCVP_id** | | **WCVP_url** | | **Location** | | **Coordinates; Altitude (m)** | | **Collection date** | | **Collector** | | **Voucher** | | **DNA extract: tissue sample** | | **Type of study** | | **Mol. Data** | | **NCBI ID** | | **Article(s) (DOI)** |
| --- | --- | --- | --- | --- | --- | --- | --- | --- | --- | --- | --- | --- | --- | --- | --- | --- | --- | --- | --- | --- | --- | --- | --- | --- | --- | --- | --- | --- | --- | --- | --- | --- | --- | --- | --- | --- | --- |
| *Rivasmartinezia vazquezii* | | Magnoliopsida: Apiales | | Apiaceae | | *Rivasmartinezia* Fern.Prieto & Cires (2014) | | *Rivasmartinezia vazquezii* | | Fern.Prieto & Cires (2014) | | --- | | --- | | 2106990 | | https://wcvp.science.kew.org/taxon/77144571-1 | | La Redibobia, Somiedo (Asturias, Spain) | | N 438 050, W 068 12; 684 | | VMV, EC & JAFP | | FCO32667 | | RV1: RV1 | | PhG | | ITS, trnL (UAA),trnL-trnF | | KC676175; KC676192; KC676209 | | https://doi.org/10.1080/11263504.2013.819818 | |
| *Rivasmartinezia vazquezii* | | Magnoliopsida: Apiales | | Apiaceae | | *Rivasmartinezia* Fern.Prieto & Cires (2014) | | *Rivasmartinezia vazquezii* | | Fern.Prieto & Cires (2014) | | --- | | --- | | 2106990 | | https://wcvp.science.kew.org/taxon/77144571-1 | | La Mexiota, Somiedo (Asturias, Spain); | | N 438 040, W 068 110; 683 | | VMV, EC & JAFP | | FCO32702 | | RV2: RV2 | | PhG | | ITS, trnL (UAA),trnL-trnF | | KC676176; KC676193; KC676210 | | https://doi.org/10.1080/11263504.2013.819818 | |
| *Micranthes stellaris* | | Magnoliopsida: Saxifragales | | Saxifragaceae | | *Micranthes* | | *Micranthes stellaris* | | (L.) Galasso, Banfi & Soldano (2005) | | --- | | --- | | 984426 | | https://wcvp.science.kew.org/taxon/77068348-1 | | Alto Campoo (Cantabria, Spain) | | 30T  386491  4766936 | | MS, EC & JAFP | | FCO32853 | | Mste) | | PhG | | ITS, rbcL | | KC749991; KC74998 | | https://doi.org/10.1007/s10265-013-0566-2 | |
| *Cochlearia pyrenaica* | | Magnoliopsida: Brassicales | | Brassicaceae | | *Cochlearia* L. (1753) | | *Cochlearia pyrenaica* | | DC. (1821) | | --- | | --- | | 103522 | | https://wcvp.science.kew.org/taxon/281484-1 | | Lontzenerbach, Wallonia (Kelmis, Liège, Belgium) | | N 50°41′, E 05°59′ | | EC & MSS | | FCO numbers: 32169–32173 | | LON (1-16): LON(1-16) | | GD | | AFLPs: EcoRI-AAG/MseI-CTA; EcoRI-ACT/MseI-CAC; EcoRI-AGC/MseI-CTA; EcoRI-AAC/MseI-CAT; EcoRI-AAC/MseI-CAA | | --- | | https://doi.org/10.1007/s00606-011-0500-9 | |
| *Cochlearia pyrenaica* | | Magnoliopsida: Brassicales | | Brassicaceae | | *Cochlearia* L. (1753) | | *Cochlearia pyrenaica* | | DC. (1821) | | --- | | --- | | 103522 | | https://wcvp.science.kew.org/taxon/281484-1 | | Between Oô and Lac d’Oô (Haute-Garonne, France) | | N 42°46′, E 00°30′ | | EC, JH & JAFP | | FCO numbers: 32169–32173 | | LOÔ (1-16): LOÔ (1-16) | | GD | | AFLPs: (see LON) | | --- | | https://doi.org/10.1007/s00606-011-0500-9 | |
| *Cochlearia pyrenaica* | | Magnoliopsida: Brassicales | | Brassicaceae | | *Cochlearia* L. (1753) | | *Cochlearia pyrenaica* | | DC. (1821) | | --- | | --- | | 103522 | | https://wcvp.science.kew.org/taxon/281484-1 | | Ascent to Puerto de Somiedo (Asturias, Spain) | | N 43°02′,W 06°14′ | | EC, JH & JAFP | | FCO numbers: 32169–32173 | | SOM (1-16): SOM (1-16) | | GD | | AFLPs: (see LON) | | --- | | https://doi.org/10.1007/s00606-011-0500-9 | |
| *Cochlearia pyrenaica* | | Magnoliopsida: Brassicales- | | Brassicaceae | | *Cochlearia* L. (1753) | | *Cochlearia pyrenaica* | | DC. (1821) | | --- | | --- | | 103522 | | https://wcvp.science.kew.org/taxon/281484-1 | | Col du Tourmalet, west face (Hautes-Pyrénées, France) | | N 42°54′, E 00°07′ | | EC, JH & JAFP | | FCO numbers: 32169–32173 | | TOU (1-16): TOU (1-16) | | GD | | AFLPs: (see LON) | | --- | | https://doi.org/10.1007/s00606-011-0500-9 | |
| *Cochlearia pyrenaica* | | Magnoliopsida: Brassicales- | | Brassicaceae | | *Cochlearia* L. (1753) | | *Cochlearia pyrenaica* | | DC. (1821) | | --- | | --- | | 103522 | | https://wcvp.science.kew.org/taxon/281484-1 | | Col du Between Villar de Vildas and La Pornacal (Asturias, Spain) | | N 42°54′, E 00°07′ | | EC, JH & JAFP | | FCO numbers: 32169–32173 | | VIL (1-16): VIL (1-16) | | GD | | AFLPs: (see LON) | | --- | | https://doi.org/10.1007/s00606-011-0500-9 | |

**The initials of the collectors correspond to: V.M. Vázquez (VMV), José Antonio Fernández Prieto (JAFP), M. Sanna (MS), H.S. Nava (HSN), M-S. Samain (MSS), Eduardo Cires (EC) and JH (JH).
