# Supplementary material for: Extending Specimens to Save Plant DNA: Structuring Department DNA Collections in Times of Biodiversity Loss
Source: Ecol Evol. 2026 Apr 8;16(4):e73211. doi: 10.1002/ece3.73211 (PMC13059098; doi:10.1002/ece3.73211)
Supplement: Supplementary file 2 — Table S2: Summary of the ideal metadata gathered to generate an Extended Specimens (ESs) and Extended Specimen Networks (ESNs). Ideally, this data should be added to the collection data (see Table 3). [file ECE3-16-e73211-s001.doc]

**Table S2**. Summary of the ideal metadata gathered to generate an Extended Specimens (ESs) and Extended Specimen Networks (ESNs). Ideally, this data should be added to the collection data (see Table 3).

| Metadata | Description |
| --- | --- |
| Collecting Name | This referes to the taxon name under which the sample was collected |
| Accepted Taxon | Taxon accepted name according to the World Checklist of Vascular Plants (WCVP) (Govaerts et al., 2021; Govaerts, 2023; Royal Botanic Gardens Kew, 2023) and/or scientific studies in which this sample has been used |
| Accepted Species | Species accepted name ( with year and author(s)) according to the WCVP (Govaerts et al., 2021; Govaerts, 2023; Royal Botanic Gardens Kew, 2023) and/or scientific studies in which this sample has been used |
| Accepted Genus | Genus accepted name according to the WCVP (Govaerts et al., 2021; Govaerts, 2023; Royal Botanic Gardens Kew, 2023) and/or scientific studies in which this sample has been used |
| Accepted Family | Family accepted name according to the WCVP (Govaerts et al., 2021; Govaerts, 2023; Royal Botanic Gardens Kew, 2023) and/or scientific studies in which this sample has been used |
| Accepted Class | Accepted class to which the family belongs according to the Angiosperm Phylogeny Group (APG) IV (the Angiosperm Phylogeny Group, 2016) |
| Accepted Order | Accepted order to which the family belongs according to the APG IV t (Angiosperm Phylogeny Group, 2016) |
| WCVP_id | Id number of the taxon in Plants of the World Online (POWO) as provided by the WCVP (POWO, 2025) |
| WCVP_url | URL of the taxon in Plants of the World Online (POWO) as provided by the WCVP (POWO, 2025) |
| Location | Name of the sampling site |
| Coordinates | Coordinates of the sampling site |
| Altitude | Altitud in meter above the sea level (m.a.s.l) of the sampling site |
| Collection date | Date of collection |
| Collector(s) | Name of the collector(s). This would be optimal if the collectors’ own collecting number was specified as well. |
| Voucher ID | Voucher ID number. This would be optimal if the Herbarium full name was also specified. |
| Voucher data (optimal) | This section should specify some data aoubt the herbarium voucher(s):  Full individual/Part of an individual  Reproductive structures: present/Not present |
| DNA extract ID | DNA extract ID number |
| Tissue sample ID | Tissue sample ID number |
| Type of study | This section should specify what type ofwas conducted with this sample: phylogenetic or phylogenomic studies (PhG); Genetic Diversity studies (GD) |
| Type of sampling scheme (optimal) | This section should specify the type of sampling of the conducted: taxonomic sampling, genetic diversity sampling or distribution validation sampling. |
| Molecular Data | This should specify what type of molecular data was obtained from the sample: whole genome (specify the DNA amplification method), barcoding (specify DNA sequences obtained), microsatellites (specify which one(s))… |
| NCBI ID | NCBI ID number(s) of the data derived from the sample |
| Article(s) (DOI) | Full citation of the article(s) where this sample has been used an their DOI(s). |

**References**

Govaerts, R. 2023. WCVP: World Checklist of Vascular Plants, Version 12. Royal Botanic Gardens, Kew. <https://sftp.kew.org/pub/data-repositories/WCVP> (accessed 19 Dec 2025).

Govaerts, R., Nic Lughadha, E., Black, N., Turner, R. & Paton, A. 2021. The World Checklist of Vascular Plants, a continuously updated resource for exploring global plant diversity. *Sci. Data* 8: 1–10. <https://doi.org/10.1038/s41597-021-00997-6>

Royal Botanic Gardens Kew. Plants of the World Online (POWO). 2025. Facilitated by the Royal Botanic Gardens, Kew. http://www.plantsoftheworldonline.org/ (accessed 19 Dec 2025).

The Angiosperm Phylogeny Group. 2016. An update of the Angiosperm Phylogeny Group classification for the orders and families of flowering plants: APG IV. *Bot. J. Linn. Soc.* 181: 399–436. <https://doi.org/10.1111/boj.12385>
